# Supplementary material for: Quantitative imaging of lipid transport in mammalian cells
Source: Nature. 2025 Aug 20;646(8084):474–82. doi: 10.1038/s41586-025-09432-x (PMC12507682; doi:10.1038/s41586-025-09432-x)
Supplement: Supplementary file 2 — Reporting Summary [file 41586_2025_9432_MOESM2_ESM.pdf]

nature portfolio

Corresponding author(s): André Nadler

Last updated by author(s): Jun 17, 2025

# Reporting Summary

Nature Portfolio wishes to improve the reproducibility of the work that we publish. This form provides structure for consistency and transparency in reporting. For further information on Nature Portfolio policies, see our Editorial Policies and the Editorial Policy Checklist.

## Statistics

For all statistical analyses, confirm that the following items are present in the figure legend, table legend, main text, or Methods section.

| n/a | Confirmed | |
|---|---|---|
| ☐ | ☒ | The exact sample size (*n*) for each experimental group/condition, given as a discrete number and unit of measurement |
| ☐ | ☒ | A statement on whether measurements were taken from distinct samples or whether the same sample was measured repeatedly |
| ☐ | ☒ | The statistical test(s) used AND whether they are one- or two-sided<br>*Only common tests should be described solely by name; describe more complex techniques in the Methods section.* |
| ☒ | ☐ | A description of all covariates tested |
| ☒ | ☐ | A description of any assumptions or corrections, such as tests of normality and adjustment for multiple comparisons |
| ☐ | ☒ | A full description of the statistical parameters including central tendency (e.g. means) or other basic estimates (e.g. regression coefficient) AND variation (e.g. standard deviation) or associated estimates of uncertainty (e.g. confidence intervals) |
| ☐ | ☒ | For null hypothesis testing, the test statistic (e.g. *F*, *t*, *r*) with confidence intervals, effect sizes, degrees of freedom and *P* value noted<br>*Give P values as exact values whenever suitable.* |
| ☒ | ☐ | For Bayesian analysis, information on the choice of priors and Markov chain Monte Carlo settings |
| ☒ | ☐ | For hierarchical and complex designs, identification of the appropriate level for tests and full reporting of outcomes |
| ☐ | ☒ | Estimates of effect sizes (e.g. Cohen's *d*, Pearson's *r*), indicating how they were calculated |

*Our web collection on statistics for biologists contains articles on many of the points above.*

## Software and code

Policy information about availability of computer code

| | |
|---|---|
| Data collection | Olympus cellSens Dimension (Version 4.1), ChipSoftManager 8.3.1.1018, Thermo Scientific Q Exactive 2.9, Bruker TopSpin 3.6.2, Thermo Scientific Xcalibur 4.4.16.14, FTMS Booster Control Software 2018.6.0, Olympus FV10-ASW 4.2, SymPho Time 64 2.6, Sepia II |
| Data analysis | Python 3.9.7 with the following packages: tifffile 2020.9.3, python-javabridge 4.0.2, python-bioformats 4.0.3, numpy 1.19.3, apeer-ometiff-library 1.8.2, pandas 1.1.5, scipy 1.5.4, scikit-image 0.17.2, scikit-learn 0.24.2, matplotlib 3.3.4, Pillow 8.4.0, opencv-python 4.7.0.72. Ilastik 1.4.Thermo Scientific Xcalibur 4.4.16.14, MestReNova 14.3. PeakbyPeak (SpectroSwiss) 2020.10.0.b1, LipidXplorer 1.2.4, Simtrim, Jupyter Notebook. ImageJ 1.54h, SymPhoTime 5.3.2.2, OriginPro 8.5, Matlab R2020b, Matlab Optimization toolbox<br><br>The code can be found at http://doi.org/21.11101/0000-0007-FCE3-D |

For manuscripts utilizing custom algorithms or software that are central to the research but not yet described in published literature, software must be made available to editors and reviewers. We strongly encourage code deposition in a community repository (e.g. GitHub). See the Nature Portfolio guidelines for submitting code & software for further information.

## Data

Policy information about availability of data

All manuscripts must include a data availability statement. This statement should provide the following information, where applicable:

- Accession codes, unique identifiers, or web links for publicly available datasets
- A description of any restrictions on data availability
- For clinical datasets or third party data, please ensure that the statement adheres to our policy

The complete lipid flux dataset can be interactively accessed under http://doi.org/21.11101/0000-0007-FCE5-B and the original data can be downloaded from http://doi.org/21.11101/0000-0007-FCE4-C and https://doi.org/10.6019/S-BIAD1695. A demo-dataset is available at https://doi.org/10.17617/3.BRSGLA. The source data for all the plots presented in the main and extended data figures is provided as separate Excel files.

## Research involving human participants, their data, or biological material

Policy information about studies with human participants or human data. See also policy information about sex, gender (identity/presentation), and sexual orientation and race, ethnicity and racism.

| | |
|---|---|
| Reporting on sex and gender | n/a |
| Reporting on race, ethnicity, or other socially relevant groupings | n/a |
| Population characteristics | n/a |
| Recruitment | n/a |
| Ethics oversight | n/a |

Note that full information on the approval of the study protocol must also be provided in the manuscript.

# Field-specific reporting

Please select the one below that is the best fit for your research. If you are not sure, read the appropriate sections before making your selection.

☒ Life sciences ☐ Behavioural & social sciences ☐ Ecological, evolutionary & environmental sciences

For a reference copy of the document with all sections, see nature.com/documents/nr-reporting-summary-flat.pdf

# Life sciences study design

All studies must disclose on these points even when the disclosure is negative.

| | |
|---|---|
| Sample size | No sample size calculation was performed. The low variability between the different labeling conditions shows that a small number of independent repeats is sufficient. Mass spectrometry measurements contained 3 biological replicates and 3 technical replicates. For microscopy experiments, at least 5 fields of view were acquired per condition to ensure statistical power for analysis. |
| Data exclusions | Images were excluded if the acquisition process failed by either being out of focus or if unwanted cellular debris or clamps precluded image analysis. No data was excluded from mass-spectrometry. |
| Replication | Mass spectrometry experiments were carried in 3 biological replicates with 2 technical replicates. Microscopy experiments for U2OS cells that were used to calculate the amount of lipid in the plasma membrane were replicated 3 times with at least 10 fields of view; ER experiments were replicated 2 times with at least 10 fields of view; Endosomes, Mitochondia and Golgi were measured once with at least 5 fields of view. Microscocopy experiments using HCT116 involving WT and TMEM30A mutants were replicated at least 2 times with at least 10 fields of view. Drug experiments to block endocytosis were performed once and at least 10 fields of view were captured. |
| Randomization | Images were taken at random positions in the wells of a 96 well-plate. For this we made use of the CellSens software. Mass spectrometry experiments cannot be randomized, the lipidome of the whole sample was quantified. |
| Blinding | Not relevant for this study. The microscopy data was acquired in an unbiased way. For mass spectrometry the whole lipidome of the sample was studied |

# Reporting for specific materials, systems and methods

We require information from authors about some types of materials, experimental systems and methods used in many studies. Here, indicate whether each material, system or method listed is relevant to your study. If you are not sure if a list item applies to your research, read the appropriate section before selecting a response.

## Materials & experimental systems

| n/a | Involved in the study |
| --- | --- |
| ☐ | ☒ Antibodies |
| ☐ | ☒ Eukaryotic cell lines |
| ☒ | ☐ Palaeontology and archaeology |
| ☒ | ☐ Animals and other organisms |
| ☒ | ☐ Clinical data |
| ☒ | ☐ Dual use research of concern |
| ☒ | ☐ Plants |

## Methods

| n/a | Involved in the study |
| --- | --- |
| ☒ | ☐ ChIP-seq |
| ☒ | ☐ Flow cytometry |
| ☒ | ☐ MRI-based neuroimaging |

# Antibodies

Antibodies used

Rab5 (rabbit), Cell Signaling Technology CST-3547S, Clone: C8B1, Lot: 7
Rab7 (rabbit), Cell Signaling Technology CST-9367S, Clone: D95F2, Lot: 3
Tom20 (mouse), Santa Cruz sc-17764, Clone: F-10, Lot: H0320
ATP5A (mouse), Abcam ab14748, Clone: 15H4C4, Lot: 2101038509
Golgin97 (rabbit), Abcam ab84340, Clone:Polyclonal, Lot:1
Giantin (rabbit), Abcam ab80864, Clone: Polyclonal, Lot:GR3209923-1
GM130 (rabbit), Cell Signaling Technology CST-12480S, Clone:D6B1 Lot: 3
Lamp1 (rabbit), Cell Signaling Technology CST-9091S, Clone: D2D11 Lot: 6
Tom20 (rabbit), Cell Signaling Technology CST-42406S, Clone:D8T4N Lot: 4
ATP5A (rabbit), Abcam ab176569, Clone: EPR13030(B), Lot:GR3291066-12
Calnexin 1 (mouse) Abcam ab112995, Clone: 6F12BE10, Lot: GR3246794-6
Lamin A/C (mouse), Cell Signaling Technology CST-4777S, Clone:4C11 Lot:5
BAP31 (mouse), Enzo Life Sciences ALX-804-601-C100, Clone: A1/182 Lot: L15093
Calreticulin (mouse), Abcam ab22683, Clone: FMC 75, Clone: Lot:GR3361946-5

Validation

All primary antibodies were used directly from the manufacturer. All antibodies were validated by the manufacturers to be suitable for immunofluorescence (IF) and reactive with Human samples.

Rab5, CST-3547S -> Applications: WB, IF Species Reactivity: Human, Mouse, Rat, Monkey
Rab7, CST-9367S -> Applications: WB, IP, IF. Species Reactivity: Human, Mouse, Rat, Monkey
Tom20, sc-17764 -> Applications: WB, IP, IF, IHC(P), ELISA. Species Reactivity: Human, Mouse, Rat
ATP5A, ab14748 -> Applications: WB, IF, IHC(P), FC. Species Reactivity: Human, Rat, Cow, Mouse, Drosophila melanogaster.
Golgin97, ab84340 -> Applications: IF, HC(P), ICC. Species Reactivity: Human
Giantin, ab80864 -> Applications: WB, IF, ICC, IHC(P). Species Reactivity: Human
GM130, CST-12480S -> Applications: WB, IF, IP. Species Reactivity: Human, Monkey
Lamp1, CST-9091S -> Applications: WB, IF, IP, IHC, FC. Species Reactivity: Human, Monkey
Tom20, CST-42406S -> Applications: WB, IF, IHC, IP. Species Reactivity: Human, Mouse, Rat, Monkey
ATP5A, ab176569 -> Applications: WB, IF, ICC, IHC(P), FC. Species Reactivity: Human, Mouse, Rat
Calnexin, ab112995 -> Applications: WB, IF, ICC, IHC(P), FC. Speacies Reactivity: Human
Lamin A/C, CST-4777S -> Applications: WB, IF, IP, IHC, IP. Species Reactivity: Human, Mouse, Rat, Monkey
BAP31, ALX-804-601-C100 ->Applications: WB, IF, IP, ICC, FC, ELISA, IHC. Species Reactivity: Human, Monkey
Calreticulin, ab22683 -> Applications: IF, ICC, FC, IHC(P). Species Reactivity: Human

# Eukaryotic cell lines

Policy information about cell lines and Sex and Gender in Research

Cell line source(s)

U-2 OS cells were purchased from ATCC and were originally obtained from a female. HCT-116 cells were purchased from ECCAC and have a male origin.

Authentication

U-2 OS cells were authenticated by eurofins genomics using 16 independent PCR-systems. HCT-116 cells were not authenticated after purchase.

Mycoplasma contamination

All cell lines are routinely tested for Mycoplasma contamination. Data presented in this study was collected only from negative samples.

Commonly misidentified lines
(See ICLAC register)

No commonly misidentified cell lines were used in this study.

## Plants

| | |
|---|---|
| Seed stocks | *Report on the source of all seed stocks or other plant material used. If applicable, state the seed stock centre and catalogue number. If plant specimens were collected from the field, describe the collection location, date and sampling procedures.* |
| Novel plant genotypes | *Describe the methods by which all novel plant genotypes were produced. This includes those generated by transgenic approaches, gene editing, chemical/radiation-based mutagenesis and hybridization. For transgenic lines, describe the transformation method, the number of independent lines analyzed and the generation upon which experiments were performed. For gene-edited lines, describe the editor used, the endogenous sequence targeted for editing, the targeting guide RNA sequence (if applicable) and how the editor was applied.* |
| Authentication | *Describe any authentication procedures for each seed stock used or novel genotype generated. Describe any experiments used to assess the effect of a mutation and, where applicable, how potential secondary effects (e.g. second site T-DNA insertions, mosiacism, off-target gene editing) were examined.* |
